# Quality Improvement Education in the Era of COVID-19: A Pivot Toward Virtual Education

**DOI:** 10.1097/pq9.0000000000000418

**Published:** 2021-06-23

**Authors:** Jessica A. Cronin, Anit Saha, Sopnil Bhattarai, Alia Fink, Lisbeth Fahey, Rahul Shah

**Affiliations:** From the Division of Quality and Safety, Children’s National Hospital, Washington, D.C.

The COVID-19 pandemic stopped healthcare in our tracks. The pandemic thrust us into different ways of caring for and protecting patients and employees. The pandemic also exposed unacceptable disparities in the management of healthcare in both microsystems and macrosystems. Providing patient care in the context of an ongoing pandemic highlighted the dire need for adaptability, the transformation of care, and the broad adoption of quality improvement (QI) methodologies to optimize healthcare delivery and safety. There are multiple examples of organizations leveraging QI tools in the COVID-19 era.^1–4^ Though definitive data are not yet available, we posit that systems that could rapidly pivot how they think about and manage care and personal protective equipment were better positioned to meet this unprecedented healthcare challenge than organizations that were slower to adapt. QI education needed to pivot too.

Children’s National Hospital, a free-standing, tertiary care, academic medical center in Washington, D.C., started a formalized QI educational program in 2015 by having multidisciplinary professionals attend Nationwide Children’s Hospital’s 4-month QI Essentials (QIE) didactic and experiential course in Columbus, Ohio. Based on the Institute for Healthcare Improvement’s Model for Improvement, QIE has an exemplary track record.^5^ We built a QI educational program within our institution called the QI Leadership Training (QuILT) Course, modeled after both of those programs and launched in 2019. QuILT incorporates in-person didactics, interactive small-group sessions, and one-on-one coaching. In addition to gaining knowledge in QI methodology, participants complete a practical component involving a QI project in their clinical or work area. This curriculum builds capability by developing a cadre of QI specialists and clinical, administrative, and executive leaders well-versed in QI throughout the organization. The first QuILT cohort had 9 participants; the second cohort started in January 2020 with 15 participants.

In March 2020, the COVID-19 pandemic reached the United States. We were suddenly faced with questions about QuILT: Do we continue? If so, how? Can the course still include an experiential component? The answer was unanimous and clear: more than ever, QI education was necessary given the importance of QI methodology to enable fast, safe, and effective healthcare delivery during the uncertainty of the COVID-19 pandemic.

Although keeping patients and employees safe necessitated limiting the number of individuals on the hospital campus, we persevered with synchronous QI education—virtually. The didactic component moved to a videoconferencing platform (Zoom by Zoom Video Communications, San Jose, Ca., zoom.us). To ensure participant engagement and collaboration that are crucial to QI education, we reviewed resources outlining tools for effective e-learning and virtual teams, and QuILT leaders coached every presenter in using these video conferencing capabilities, including instant messaging, polling, and break-out rooms.^6,7^ Throughout formal talks, presenters still had frequent check-ins with participants. Participants answered polling questions, annotated on a shared screen, and reacted to specific aspects of a presentation through instant messaging. Break-out rooms offered a structured, small-group environment that addressed specific agendas and fostered meaningful interactions, including relationship building. Before the pandemic, learners were required to complete a QI project in their clinical or work area, with step-by-step coaching from the QuILT leadership team (Table 1).

**Table 1.** Changes in Modalities with Transition from In-person to Virtual Curriculum

Modality	In-Person Curriculum Pre-COVID-19	Virtual Synchronous Curriculum Post-COVD-19
Didactic/classroom	• Frequent cooperative dialog between presenter and participants throughout formal presentations • Frequent break-out small-group sessions	• Required participant and speaker video and audio presence • Regular, purposeful check-ins between presenter and participants throughout all formal presentations, including: ∘ Polling questions with required 100% response rate from participants ∘ Reactions and comments regarding specific aspects of the presentation shared through instant messaging ∘ Annotation by all participants on a shared screen • 1–2 virtual break-out periods during every session with specific agenda to enhance small-group interaction between participants and a facilitator
Practical workshop (for data management, analysis, and presentation)	• Small-group sessions with participants on computers with their data or dummy data	• Small-group sessions with social distancing or one-on-one sessions with screen sharing
Coaching 1:1 Sessions (by prior QuILT/QIE graduates)	• In person and on phone as needed throughout course	• In person, on phone, and videoconference based on need
Individual consultation (by performance improvement consultants)	• In person and on phone as needed throughout course	• In person, on phone, and videoconference based on need • Support tools for conducting QI project team sessions on videoconferencing platform as needed

There were challenges. Presenters had difficulty appreciating visual cues indicating learner reactions and understanding. Learners started the course with varying abilities navigating the virtual platform. Unstructured interactions among participants, QuILT team members, and speakers were more limited. The experiential component was also challenging as clinical situations changed dramatically due to the pandemic. Like most hospitals, Children’s National saw decreased patient volumes in specific service lines, which directly impacted many QI projects.

Nonetheless, many participants completed their original QI projects. One participant implemented a process to identify deteriorating patients early and reduce nonintensive care unit arrests, which is crucial even when the patient census is decreased. Others adjusted their projects to address newly identified needs, like improving telemedicine access. Regardless of the experiential project, we coached participants in QI methodology and supported them as they faced unique challenges doing QI work amidst the COVI-19 pandemic. For example, participants learned to use videoconference capabilities, such as annotation on shared screens, to develop key driver diagrams, process charts, and other QI tools with their teams.

We were able to provide the same components of the course virtually as we did in-person. Like the in-person cohorts, this “virtual cohort” spent 33 training hours learning about QI science through didactic and experiential learning. But was it comparable in quality? We evaluated participants’ precourse and postcourse scores in competency domains, including knowledge of QI science, data management and analysis, spreading, and sustaining science. Competency gains were substantial in the cohort that underwent the virtual QI curriculum despite small sample size limitations. These gains were not significantly different than the gains experienced in the prior in-person cohort (Fig. 1). We found that, virtually or in-person, our program can successfully increase QI capability at our institution. We continue to apply lessons learned to improve our virtual QI education platform in the new COVID-19 era *until* the pandemic is resolved.

**Fig. 1.**
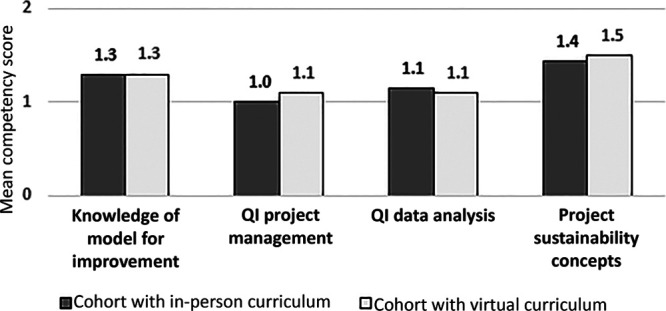
Gains in competency scores after in-person vs virtual QI education curricula. Self-assessment of participants’ QI competency for 2 cohorts who completed the QI Leadership Course (QuILT) at Children’s National. Nine participants completed the in-person curriculum in Fall 2019, and 14 participants completed the virtual curriculum in Spring 2020. “Mean Competency Score Gain” = mean increase in self-assessed competency score after the course for all respondents. Scores are based on a scale of 1–5. There was no statistical difference between mean competency score gains between cohorts based on a threshold of *P* < 0.05 by the Wilcoxon sign-rank test.

This commentary is a firm stance that the show must go on. We share our pivot to virtual education, celebrate our team’s achievement in finishing the course, and support QI leaders’ ongoing development with hopes that similar organizations can generalize from our early learnings. QI capability-building via education is imperative during normal times; during a pandemic, it is crucial.

## DISCLOSURE

The authors have no financial interest to declare in relation to the content of this article.
